# Creativity in the Here and Now: A Generic, Micro-Developmental Measure of Creativity

**DOI:** 10.3389/fpsyg.2018.02095

**Published:** 2018-11-08

**Authors:** Elisa Kupers, Marijn Van Dijk, Andreas Lehmann-Wermser

**Affiliations:** ^1^Department of Special Needs Education and Youth Care, University of Groningen, Groningen, Netherlands; ^2^Department of Developmental Psychology, University of Groningen, Groningen, Netherlands; ^3^Institut für Musikpädagogische Forschung, Hochschule für Musik, Theater und Medien Hannover, Hanover, Germany

**Keywords:** creativity, microgenetic theory, process research, observational methods, scientific reasoning, musical composition

## Abstract

Creativity is a relevant yet elusive concept, and consequently there is a large range of methods to assess creativity in many different contexts. Broadly speaking, we can differentiate between creativity measures on the level of the person (such as the Torrance tests), the level of the creative product (consensual assessment), and the level of the creative process. In the recent literature on children's creativity, 80% of the studies employed measures on either the person or the product level (Kupers et al., submitted). However, for parents, teachers, and employers who wish to stimulate creativity, insight in the (often socially embedded) creative process is badly needed. This move from the inter-individual to the intra-individual level of assessment is furthermore in line with research in many other domains in psychology. Although there is some research focusing more on detailed descriptions of creative processes, the studies are usually purely qualitative and therefore highly context-specific, making generalization difficult. In this paper, we present a newly developed coding frame as a systematic, generic, micro-level measure of creativity. What is unique about this coding frame is that it can be applied to observations of creative processes in many different contexts, and for different kinds of creative tasks. The core of the instrument is that it allows us to assess the two core components of creativity - novelty and appropriateness on an ordinal 4-point scale, at each moment during the creative process. The coding frame can be applied in three steps. The first step is to determine the unit of analysis, that is, the level of detail in which the creative process is assessed. The second step and third steps are coding the units on two ordinal scales of novelty and appropriateness, respectively. In order to illustrate the versatility of our instrument, we apply it to two cases of very different creative processes: a musical composition task (open-ended) and a scientific reasoning task (closed- ended). Last, we demonstrate the possibilities for analyzing this type of dense intra-individual measurements of creativity (time series analysis and state space grids) and discuss the future research that is needed to fully validate the instrument.

## Introduction

Creativity is the human capacity to use your imagination and create to create solutions for complex problems (Welch and McPherson, 2012). Therefore, it is essential for our survival and prosperity. Creativity has been recognized as one of the most important “twentieth century skills,” which should be leading in shaping current and future educational policy and practice. For teachers, managers, and others who wish to stimulate creativity, it is therefore important to gain an understanding of how creative processes unfold in the here and now.

In the study of human behavior, there is currently an increasing interest in real-time processes relating to fundamental issues such as intra-individual variability as a mechanism of change. These developments are being further enhanced by technological advancements that make the collection of dense intra-individual data more feasible, such as EMA (ecological momentary assessment: Shiffman et al., 2008), eye-tracking, (e.g., Odean et al., 2015), and wireless heart rate monitoring (e.g., Vickhoff et al., 2013; Gregersen et al., 2014). Within empirical research on creativity, however, processes in the here and now are often overlooked—possibly due to a lack of systematic, quantitative measurement instruments that can be used for measuring creativity across a variety of contexts. In this article, we will explain both why such an instrument is indispensable and which criteria it needs to meet. We will present a basic coding frame for assessing the key elements of creative processes (novelty and appropriateness), and will use two empirical examples to illustrate how this framework can be applied. Furthermore, we will describe the steps involved in applying the framework to two particular cases of creative behavior (during an open-ended musical composition task and during a closed-ended scientific reasoning task). We conclude with implications for creativity research and the next steps needed in order to further validate the instrument.

## Creativity as novelty and appropriateness

Creativity is defined as “the interaction among aptitude, process, and environment by which an individual or group produces a perceptible product that is both novel and useful as defined in a certain social context” (Plucker et al., 2004, p.90). On the one hand, creativity is something unexpected; something beyond what is already known at a certain point. On the other hand, the definition implies that creativity requires more than just novelty; the response or product must also be useful or appropriate (Cropley, 2006; Runco and Jaeger, 2012). It must be a fitting solution to the task or problem at hand. The characteristics of novelty and appropriateness relate to the two distinct processes that together make up creativity. The first process is *divergent thinking*, which is the skill to generate as many possible solutions to a problem as possible. Divergent thinking requires a person to be able to associate quickly, make unexpected links between components, and transform information into unexpected forms (Guilford, 1967a; Runco, 2010). Three features of divergent thinking are usually assessed: fluency, flexibility, and originality (Guilford, 1957; Sternberg, 2006; Baas et al., 2008). Fluency refers to the amount of unique ideas a person is able to generate within a fixed amount of time. Flexibility is the capacity to be able to quickly switch between approaches to and characteristics of the problem at hand. Consider the example where a child is asked to come up with as many uses of a paperclip as possible. One child may respond: a paper binder, a necklace, a tool to open a lock. Another child may respond: a necklace, a bracelet, earrings. Although the fluency of these two sets of responses is the same, the second child demonstrates a lower level of flexibility as each solution stems from the same overall semantic category (jewelry). The third component, originality, refers to the uniqueness of an idea or solution. When comparing children's responses to the “paperclip problem,” some responses might be very common (such as the “paper binder” response) while others are more uncommon (such as the “tool to open a lock” response). In creativity research, divergent thinking is often equated with creative thinking. However, as previously mentioned, divergent thinking entails more than just novelty; usefulness or appropriateness is also important. For true creativity, we need to evaluate whether the many solutions generated contribute in any way to solving the problem or finishing the task at hand. This involves *convergent thinking*. While divergent thinking is the generation of as many solutions to a problem as possible, convergent thinking is defined as “oriented toward deriving the single best (or correct) answer to a clearly defined problem” (Cropley, 2006, p. 391). Convergent thinking is closely connected to using prior knowledge; in order to arrive at the best solution to a problem, one must know what is already known about the problem and build on that existing knowledge. A problem has certain aspects or constraints, and being able to deal with these task constraints is what eventually determines whether an idea is actually creative (Cropley, 2006; Glǎveanu, 2013b, 2014). This applies across domains, from clearly defined scientific problems, to literature, poetry, or music (Cropley, 2006). In cognitive models of creativity, divergent, and convergent thinking are often closely interlinked. Within the theory of blind variation and selective retention (BVSR) for example, divergent thinking plays a role in ideation or the generation of possible ideas, convergent thinking mainly in the selection of fruitful ideas (Simonton K., 1999; Simonton D. K., 1999; Simonton, 2015).

## Assessing creativity

When looking at how creativity can be assessed, a distinction can be made between three levels on which creativity can be measured: the level of the person, the level of the product, and the level of real-time actions. A similar distinction is made in Rhodes “4P” model of creativity (see Rhodes, 1961), where he distinguishes between creativity on the levels of the Person, Product, Process, and Press (the latter referring to environmental influences). These levels of measurement differ in the extent to which they see creativity as an aggregated construct—for instance, as the average across moments, products or even a person's lifetime (Kupers et al., submitted). The highest level of measurement is the level of the **person**. Here, creativity is seen as a personal characteristic that may or may not change over time. Assessments of creativity on this level can answer questions about differences between groups of people—for instance between men and women (Baer and Kaufman, 2008), between cohorts of different generations (Kim, 2011), or between children with and without developmental disorders (Healey and Rucklidge, 2006; Tafti et al., 2009; Kim and VanTassel-Baska, 2010). Alternatively, questions can be answered about the relation between creative thinking and other personal variables, such as IQ. The most frequently used assessments on this level are tests for divergent thinking, such as the “Guilford Alternative Uses test” (Guilford, 1967b) or the “Torrance Tests of Creative Thinking” (Torrance, 1966). These types of tests come in many different forms, but they all involve asking someone to come up with many different responses to a single problem. This can be a verbal task—such as when someone is asked to come up with as many alternative uses of a brick as possible—or a non-verbal task—for instance, completing a drawing based on one shape. The extent to which a person is then considered creative depends on how their responses score for flexibility, fluency, and originality. Some of these divergent thinking tasks (such as the Torrance Test of Creative Thinking) also take into account a score for elaboration (see for instance Torrance, 1966).

In the past few decades of creativity research, the most prominent way of assessing creativity has been through divergent thinking tasks (Long, 2014; Kupers et al., submitted). Another type of creativity test is formed by problem-solving tasks, in which one specific way of solving the problem tends to be considered the “correct” response. For this reason, these types of tests mainly assess convergent thinking. Some less commonly used measures of creativity on the level of the person include personality tests or interviews, either self- or other assessments ( e.g., Runco et al., 2001; Butcher and Niec, 2005; Kaufman et al., 2010; Putwain et al., 2012). In the domain of self-report questionnaires, a distinction can be made between self-reported creative achievements or behaviors on the one hand (participants rating whether they wrote a book, achieved success in an artistic domain, etc.), questionnaires or interviews of creative self-concept (participants' ideas about whether they view themselves as creative) on the other. Both types of self-reported creativity can be assessed in a reliable and valid way (Silvia et al., 2012). Creativity is also assessed by having others evaluate creative **products**, such as written poems or stories, musical compositions and paintings. This type of assessment acknowledges that the decision regarding “what is truly creative” is inherently intersubjective; something is creative when people who are familiar in the domain judge it as creative. These types of assessments are commonly known as “consensual assessments,” based on the work of Amabile (1983, 1996). Similarly to assessments on the person level, assessments on the product level can be used to answer questions about group differences in creativity—but they are also used to measure the effect of (educational) interventions (e.g., Patera et al., 2008).

On the level of real-time actions, studies zoom in on the creative **process** as it occurs in the behaviors of individuals from moment to moment. These types of studies aim to get more insight into things like how the creative process unfolds, whether a distinction can be made between different “stages” of the creative process, etc. (e.g., Burnard and Younker, 2004). The creative processes that are studied can be either individual or more socially situated. Studies on social creativity are focused on questions of whether and how social interactions, such as interactions between peers or with a teacher, help to shape creativity (e.g., Vass, 2007; Fernández-Cárdenas, 2008; Chappell and Craft, 2011; Glǎveanu, 2013a). In the “Four Ps” model of creativity, these environmental influences are referred to as “**press**” (Rhodes, 1961). The data in these studies on socially situated creativity are almost always qualitative—such as video observations or field notes, which are coded “bottom-up” to make sense of the data. In a systematic review of empirical literature on children's creativity published in the last decade (Kupers et al., submitted), we found that the vast majority of papers (80%) assessed creativity either on the person level or on the product level, as described above. This is in line with earlier work by Long (2014). Although his categorization system is slightly different, we can conclude that in the last two decades creativity research has shifted—from largely qualitative process descriptions of creativity, toward largely quantitative descriptions of creativity being quantitatively by means of creativity tests. This type of quantitative research, which assesses creativity on a more aggregated level (the level of the person), has provided valuable insights into group differences in overall creativity (e.g., Baer and Kaufman, 2008; Cheung and Lau, 2010). Moreover, these measures are often used to evaluate the effect of (educational) interventions targeting creativity (e.g., Hu et al., 2013; Dziedziewicz et al., 2014). Then again, the danger of focusing on creativity on these aggregated levels is that the core of creativity, namely the creative process (Glǎveanu, 2013b; Kupers et al., submitted), is overlooked. Qualitative studies on the process level of creativity have offered rich, detailed descriptions of many different types of creative processes. However, due to the type of analysis used—which is intrinsically qualitative, ethnographic, and “bottom-up”—it is very difficult to generalize any findings beyond their original context, or to test hypotheses regarding different kinds of processes or conditions.

In order to measure creativity in real time, there must be a focus on (real-time) behavior in the “here and now” in a specific context. Such a measure would enable us to describe the “microdevelopment” of creativity: the development of creativity that unfolds during a short time span (days, hours, minutes). A micro-developmental study takes the changing individual—together with his or her immediate social and physical environment, such as the interaction between a child, teacher and task—as the fundamental unit of analysis (Granott and Parzialle, 2002; Lavelli et al., 2005). For this purpose, micro-developmental studies use dense observations and employ intensive analyses to capture the processes of change (Siegler and Crowley, 1991; Granott et al., 2002). Many studies that look into micro-developmental changes during a task stem from the domain of cognitive development (such as Siegler and Chen, 1998), but the term is also used in studies within other domains—such as problem-solving (Chen and Siegler, 2000), mother-infant communication (Lavelli and Fogel, 2002), early emotional development (de Weerth et al., 1999), and second language acquisition (Sun et al., 2016). Micro-developmental data are more detailed than data collected through other methods, and can be used to analyze trial-by-trial variability, detect transitions, and analyze instructional manipulations (Siegler, 2002). The idea behind this approach is to examine changes as they are occurring (Siegler and Crowley, 1991). Gaining this type of knowledge about creative processes is of crucial importance for theory building, and indispensable for anyone who wishes to stimulate creativity. In order to take the field of creativity research a step further, an instrument is needed that enables researchers to assess creativity on the level of the creative process as it unfolds from moment to moment, in the here and now. This instrument should preferably be applicable to many different contexts, thereby making it possible for researchers to compare contrasting processes and to draw conclusions about individual differences. In the remainder of this article, we present such an instrument. The method we propose has its roots in qualitative methodology of systematic coding (Gläser and Laudel, 2013) and qualitative research into individual and social creativity. However, the proposed method is new in the sense that it *quantifies* qualitative data on two ordinal scales. This enables the micro-developmental analysis of patterns within creative behavior. Some specific options regarding this type of analysis are also presented in the remainder of this article.

## A generic micro-developmental coding framework of creativity

If we aim to measure creativity on a “real-time” level—that is, as creativity occurs in the here and now—we need to focus on both aspects of its definition: novelty and appropriateness. In the next section, we will describe three necessary steps toward constructing a generic coding scheme tailored to meet the needs of specific contexts in which creativity is measured. It is important to note that what we are offering here is a *framework* (including guidelines) for coding creative processes, which researchers can use to construct their own coding schemes. For that reason, we will present a detailed illustration of how the coding framework can be applied and tailored to specific data.

### Step 1: determine the unit of analysis

When assessing creativity from moment to moment, the first step is to determine what those “moments” or units are. It is important to note that this decision depends on the nature of the particular creative processes being studied. For instance, when a professional artist is making a painting or a sculpture, every small variation or new idea is likely to take considerable time to prepare and execute. In this case, each “turn” can take minutes. However, in a situation where two students have to write a poem together, they may well think out loud, trying out different combinations of words, sounds and meanings in rapid succession. In this case, each turn may only take a matter of seconds. Since this can differ for different creative processes, a unit of analysis is always based on observable behavior of the individual.

In order to determine a valid unit of analysis, it is important to consider the following criteria. First, to be able to analyze trends over time within the execution of an assignment or the making of a product, the codes (which will result from the coding scheme as a whole) must be sufficiently detailed. Second, units of analysis should be on the level of ideas or variations. Again, what these ideas or variations are depends on the nature of the creative process. If the process is primarily verbal, or if the product is in written language (stories, poems, scientific reasoning, etcetera), a straightforward choice for the unit of analysis would be each verbal (spoken) turn or utterance. In this case, transcripts can be based on an existing language transcription system such as CHILDES (MacWhinney, 2000), which offers guidelines for determining utterance boundaries and turns. If the creative process is primarily non-verbal (dance, arts), the unit of analysis could be any meaningful action (within dance this might be each movement, turn or step; within visual arts it could be adding new lines or figures to a drawing or painting; within musical composition it could be each musical motive). If the creative process is primarily non-verbal, but also includes verbal elements (for instance, students working together on a musical composition and negotiating which musical motives to add to the overall composition), then turns can be either verbal (spoken turns), non-verbal (meaningful actions), or a combination of both (i.e., proposing an idea and executing it at the same time would be coded as one turn).

Determining the lowest level categories is crucial for *any* coding system, and this level should be defined both conceptually and operationally. It is recommended that researchers describe the units of analysis in conceptual terms, provide prototypical examples, and also describe non-units and examples that would not be coded as a unit (Yoder and Symons, 2010). For instance, in a specific study on creativity in the building of a tower, researchers may decide to code each time a child picks up a block, each time a block is placed, and each time a block is taken away—but *not* each time the child scratches his nose, or merely touches a block. As with any specific coding system, researchers should be trained in determining units of analysis. To make the procedure more transparent, it is also recommended for inter-observer reliability to be established on the level of unit segmentation before codes are assigned (Strijbos et al., 2006). Any disagreement between researchers can be used to refine the decision guidelines concerning unit segmentation, until reliability is satisfactory.

### Step 2: code each unit for novelty

**Figure 1:**
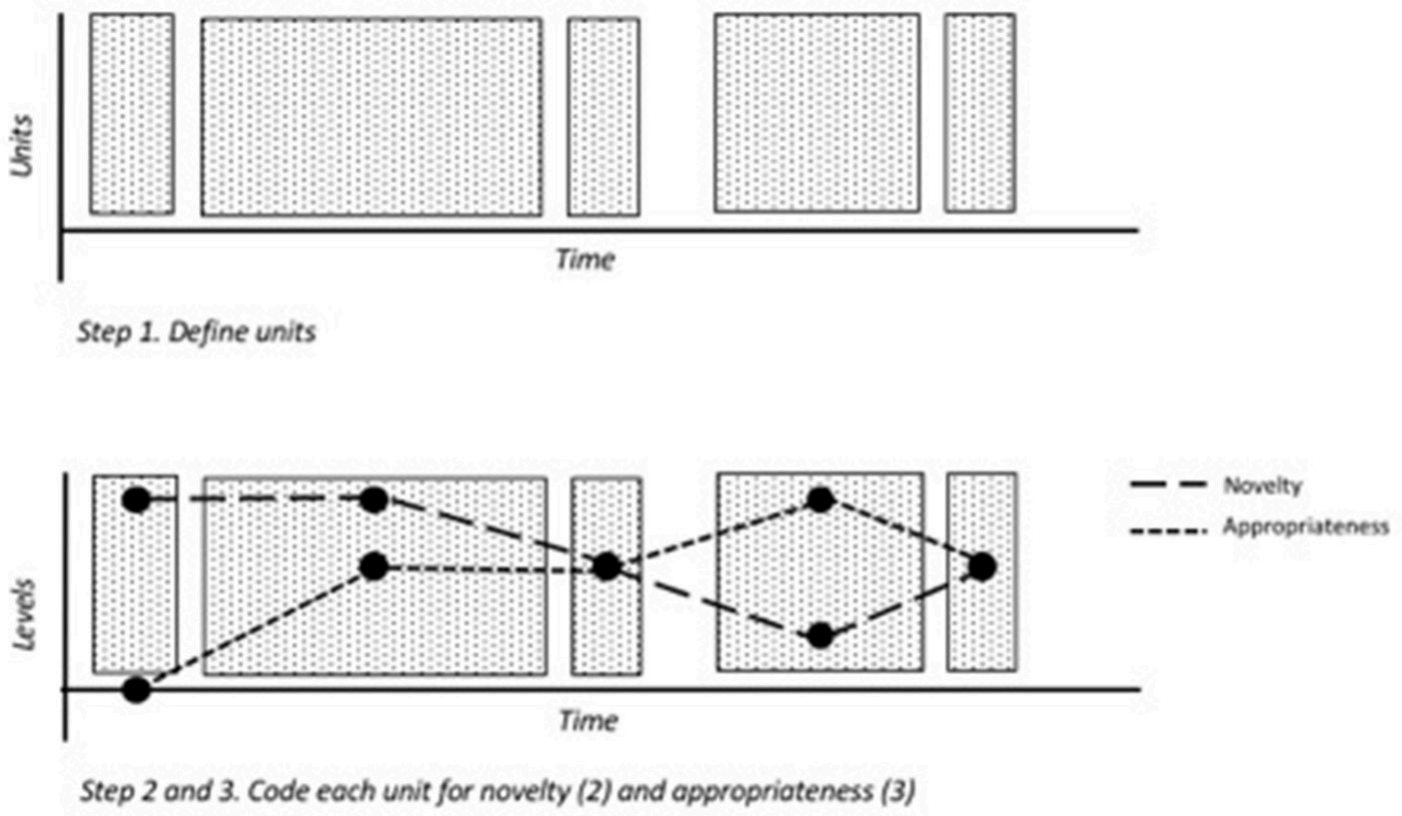
Step 1, 2, and 3: Define units (1), code each unit for novelty (2), and appropriateness (3).

Once the units of analysis have been determined, and the segmentation of the data has proven to be reliable, the next steps consist of coding each unit. This must be done on both the novelty and appropriateness dimensions (see Figure 1). These dimensions are summarized in Tables 1,2 below. Since novelty and appropriateness are relative terms (something is novel compared to what?), it is important to bear in mind that novelty is assessed on an intra-individual level—that is, *something is assessed as novel or less novel compared to what has happened up until that moment*. Importantly, this is in contrast to common divergent thinking tasks, which assess how novel a response is compared to the responses of a norm group.

**Table 1:** Coding frame for the novelty dimension.

**Level**		**Description**
0		The current turn/idea is a **repetition** of the previous turn. No new information is added. Also: a **confirmation** of the previous idea (agreeing with previous, listening back, evaluating, summarizing the process so far). A rejection of an idea without proposing an alternative.
1	The current turn is related to the previous turn, but also adds something new.	**Small elaboration:** The current idea contains subtle differences compared to the previous idea, small variations on one main idea
2		**Large elaboration:** The current idea contains more substantial differences, or multiple elements are added
3		The current turn is a **new idea**, initiative or proposition. The idea has no common elements compared to the previous turns. The child verbalizes a new component that has not been mentioned before, uses new material or performances completely novel actions upon the existing material.

**Table 2:** Coding frame for the appropriateness dimension.

**Level**	**Description**
0	**Off-task behavior:** The child walks away from the task, talks about completely unrelated subjects, or does something unrelated to the task
1	**Somewhat related to task**: The child uses task materials in a way not obviously related to task, or talks about something only remotely related to the task
2	**On-task behavior:** The child shows focused, concentrated work related to the task
3	**Explicit reference to task elements:** The child makes a link (verbal or non-verbal) with elements that are embedded in the task

The core of the novelty dimension, as described in Table 1, is assessing how much the current idea or turn has in common with the previous ideas that have already been observed. The categories are loosely based on the coding scheme of Miell and Macdonald (2000), in which a distinction is made between transactive turns (elaborating on what has previously been said or done) and non-transactive turns (either adding no new information or going in a completely new direction). When we translate this to the construct of novelty, three to four ordinal categories can be distinguished conceptually: a turn with no novel elements, a turn with partially prior elements, and partially novel elements (possibly with subcategories), and a turn with only novel elements. On the lowest level (0), the current turn adds no new elements to the turns before; it is simply a repetition or confirmation of the ideas up until this point. Regarding verbal responses, saying “I don't know” or disapproving of an idea without offering another suggestion also fall into this category. Level 1 and 2 are both elaborations, meaning the current turn builds upon previous turns (it has some common elements compared to the previous turns, but also adds something new). In most cases, a distinction can be made between small elaborations (level 1, in which only one element is added or more subtle changes are made) and large elaborations (level 2, in which more elements are added or more substantive changes are made). Level 3 means the current idea does not contain any elements that had already been mentioned.

Again, the specific descriptions of which behaviors (units of analysis) belong to which of the four categories should be described in any specific coding scheme. At this step, conceptual and operational guidelines should be established again, as should inter-observer reliability.

### Step 3: code each unit for appropriateness

The core of the appropriateness dimension is to assess how much the current turn fits the overall task or assignment. As is the case with novelty, the exact number of categories on the ordinal scale can be adjusted to the nature of the task—but on conceptual grounds, we propose an ordinal scale of at least three or, if possible, four categories. The lowest level (level 0) is off-task behavior, such as talking about unrelated topics or walking away from the task. Level 1 codes are assigned to behaviors that have some relation to the task, but use task elements in a way that is not clearly related to the task. Examples of level 1 codes would be dancing to the music when composing a musical piece on a computer, or talking about hospital syringes during a linking syringes task at school. Level 2 is assigned to behaviors that are focused and on-task, such as (in case the task is making a musical composition) browsing through a library of music loops and clicking on several to see whether they sound appealing. Finally, Level 3 is assigned to behaviors that explicitly refer to specific task elements or how to complete a task (it can contain metacognitive elements). An example of level 3 behaviors in a task where the aim is to link two syringes and make the air go from one syringe to the other, would be when the child pushes one of two connected syringes or says “Now the air goes from here to here!” As in the previous two steps, researchers of any topic should define beforehand which behaviors belong to which of the levels, train coders, and establish reliability.

## Two empirical examples

In the following section, the coding framework is applied to two case studies. These were selected as representative cases out of larger samples of video data, taken respectively from a study on musical creativity (Kupers, 2013) and a study on scientific reasoning and problem-solving (Guevara-Guererro, 2015). We present these examples here to demonstrate the steps that need to be taken in order to construct a coding scheme for coding creative behaviors in a specific task, on the basis of the framework presented in this article. For this demonstration, we chose two contexts in which the generation of new ideas plays an important role, but that were very different in other regards. The first example concerns an open-ended task in the context of music education, and the second one concerns a closed-ended task in elementary school science. The case studies serve to illustrate the potential for applying a generic measure in many different (educational) contexts. Due to the illustrative nature of these case studies, and since determining the inter-observer reliability of a coding scheme quantitatively generally requires more data than just two short cases, calculating inter-observer reliability is not appropriate in this phase of developing the framework. However, discussions did take place between the first and second author(s) regarding the segmentation and assigned codes of all data.

### Example 1: a musical composition task

#### Participants

The data of the first case study were selected from a larger study on teacher-student and peer interactions during a musical composition task in primary education (Kupers, 2013). From the six teacher-student dyads, interactions that were dominated by the teacher were considered not suitable, as sufficient student actions and utterances are required in order to fully illustrate the application of the coding framework. One of the dyads (“John” and his teacher) was selected as a case study for the current article. The video of this particular dyad gave the overall impression that the student was very much an active participant in the creative process, which is why we picked this case as most appropriate for illustrating the coding procedure. The student, a Dutch boy (native Dutch-speaking), was 9 years old at the time of data collection. The teacher was an undergraduate student in music education, who was doing a teaching internship at the student's school. The teacher and the student's parents gave their consent for participating in the study—in line with the guidelines of the Ethical Committee of the University of Groningen, department of Pedagogy and Educational Sciences.

#### Task and procedure

The assignment was to compose a short musical composition on the basis of a scene from a movie or book, using composition software. The students first had a short introduction class in which the role of music in telling a story was explained (illustrated by scenes from a Harry Potter movie), and in which the basis of the musical composition software (Magix Music Maker) was discussed. Furthermore, the teachers attended a short workshop about the basics of teaching for creativity, after which they had the opportunity to practice using the composition software. After this introduction, the student and teacher worked on the task for 30 min (using a laptop), in a room separate from the normal classroom. The software, Magix Music Maker, works with an extensive library of “loops”: short fragments of music, beats or sounds that can be selected and dragged onto a “canvas,” where the loops can be put together and edited (for instance, adjusting the dynamics or length of the loop) in order to compose a piece of music. Two video cameras were installed to record the composition process: one in front of them (facing the teacher and student) and one behind them (recording the actions on the computer screen). Participants were aware that they were being filmed. Afterwards, the spoken language was transcribed (at the level of interpretation) in F4 (transcription software), then exported to Excel where descriptions for non-verbal behavior were added with time stamps. We converted the time to timepoints of half seconds (meaning time point 10 occurs 5 s after the start of the video). These turns were then coded by using our coding frame. Both the segmentation of the data into turns (step 1) and the coding of the turns (steps 2 and 3) were extensively discussed by the first author (who coded the data) and the co-authors.

#### Application of the coding scale

##### Step 1: determine the unit of analysis

In this context—a student working on a musical composition task, supported by a teacher—we chose to only code the student turns for novelty and appropriateness (teacher turns could still be coded on other dimensions at a later point; see “Further analyses”). A turn could be either verbal, non-verbal or a combination of both, because the task entails both constructing something (a product) as well as reflecting on the actions and thinking out loud. For verbal units, each time the student made a remark, answered a question, etcetera, this was defined as a turn. In this case, turns are more suitable as units of analysis than utterances, because answers, ideas, and elaborations often encompass multiple utterances. Non-verbal turns were defined as “meaningful actions,” in the sense that they were part of the creative process, compared to merely procedural ones (e.g., saving the document, restarting the program after an error). In this context, examples of meaningful non-verbal turns were playing and selecting a loop, adjusting the volume or length of loops that were already in the composition, deleting parts of the composition, and playing back a composed piece of music. If a meaningful action was accompanied by a verbal turn (e.g., saying “I'll put this at the beginning” while dragging a loop to the beginning of the piece), they were coded together as one turn since the action and verbal turn together make up one meaningful unit. If the student voiced a new general idea that took multiple actions to execute, these “minor actions” were coded as one turn (e.g., saying “I'll make all of these very loud” and then adjusting the volume of multiple loops). Verbal turns and actions that referred to technical errors of the software or that were strictly procedural (e.g., “This loop doesn't work,” “How do you adjust the volume?”) were excluded from the analysis. In cases of doubt, the segmentation of turns was discussed by the authors. This procedure resulted in 68 turns in the first 10 min of the assignment, which were then coded for novelty and appropriateness.

##### Step 2: code each unit for novelty

The next step is to code all turns by dividing them into one of the four levels of novelty. In Table 3 below, the first part of the coded transcript is presented, accompanied with explanations for each given code.

**Table 3:** First 17 turns (9 student turns) of the musical composition task coded for novelty, translated to English by the first author.

**Turn**	**Transcript**	**Level novelty**	**Motivation**
1	*T: Do you have a scene in mind?*		
2	S: A scene, no not really.	0	Student doesn't come up with an idea yet.
3	*T: No?*		
4	S: Uh, it's hard. A scene from a movie…	0	Student doesn't come up with an idea yet.
5	*T: Or a scene from a book*.		
6	S: That's also possible. Yes, uh, well… The Grey Hunter.	3	The book “The Grey Hunter” is a new idea, it has not been mentioned before.
*7*	*T: The Grey Hunter*.		
8	S: Yes, that's very exciting.	1	The term “exciting” adds a small part of new information about the book; it refers to a nuance in the atmosphere of the book.
9.	*T: Very exciting, okay. Do you know something, anything that happened [in the book] or something?*		
10.	S: Yes, they lit a bridge on fire and then they fell off the bridge.	2	The student now goes from the general idea of the book to describing one specific scene in some detail. This is a large change compared to the previous general talk about the book.
11.	*T: Ah, well. What kind of music would go with that?*		
12.	S: Something with guitar, I think.	3	The idea that guitar could go well with the scene from the book is new.
13.	*T: Okay*.		
14.	S: And drums.	3	The idea of adding drums is new.
15.	*T: Go ahead*.		
16.	S: Guitar… [plays a guitar loop]	1	Small elaboration (trying one specific guitar loop) on the previous general idea.
17.	S: [plays another guitar loop]	1	Small elaboration (trying another specific guitar loop) on the previous general idea.

*S, Student; T, Teacher. Teacher turns are in italics. […] marks excluded, procedural turns*.

##### Step 3: code each unit for appropriateness

Step 2 was repeated, only now coding all turns for appropriateness. All turns were coded on high levels of appropriateness (level 2 and 3), meaning the student was engaged in the task during the entire fragment. Since the assignment in this case was to compose a piece of music to go with a scene from a story, level 3 was assigned when the student verbally made a link between elements of the story (events occurring in the scene, the atmosphere of the scene, etcetera) and the music. Level 2 was assigned when the student was working on-task, but without explicit referral to the task.

### Example 2: a problem-solving task about air pressure

#### Participants

The data of the second case study came from a larger study on peer interaction and scientific reasoning (Guevara et al., 2016), for which permission of the Ethical Committee of Psychology was received (ppo-011-128). For the current case we chose the interaction between one 6-year-old girl (who we will refer to as “Sarah”) and a researcher. Although Sarah was living in the Netherlands, she went to an international school and her native language was English. Therefore, the experiment was conducted in English. The researcher was a trained PhD-student. This specific case study was selected for solely pragmatic reasons: the researcher and the parents of the child gave informed consent for the use of this video, and the recording was of good technical quality.

#### Task and procedure

The task consisted of a set of tubes and syringes that had to be connected to each other in order to reach a certain goal (on one of the syringes, the plunger had get to a red mark). The overarching theme of the task was the understanding of air pressure. The syringes had different sizes and the tubes had different shapes. The child was asked to use the materials (connect materials and push the syringes) to reach the goal, and also to describe, predict and explain what was happening. The task consisted of a sequence of steps that introduced different elements. In this example, we used three elements: a first in which two equally sized syringes had to be connected, a second in which one small and one large syringe were used, and a third in which a Y-shaped tube had to be connected to two syringes. In total, this part of the task took roughly 4 min. The experiment was video-recorded, and participants were aware that they were being filmed. All spoken language and any actions involving the materials were transcribed in Excel, in which descriptions of those actions were added with time stamps (manually). It should be noted that any spoken language was transcribed at the interpretation level (meaning we corrected for grammatical errors, false starts, unintelligible parts, etcetera.).

#### Application of the coding framework

##### Step 1: determine the unit of analysis

The child's utterances and actions were considered the units of analysis, and coded for novelty and appropriateness. Considering the units of analysis, we followed CHILDES guidelines with regard to determining utterance boundaries (MacWhinney, 2000). The reason for using utterances as verbal units instead of turns is that, in this task, the aim was to form an understanding of the principles of air pressure, and separated utterances might already contain information about this understanding without being a completely formed “idea.” Actions were separated on the basis of meaningful chunks of movements: (attempts at) pushing/pulling the plunger (attempts at), connecting two elements with each other, turning an object in another direction, pointing toward an object, blowing into a tube, etcetera. In this context, meaningful units were any manipulations of the materials, such as connecting or disconnecting syringes, pushing or pulling plungers. All verbal utterances were also considered meaningful. Unintelligible language and giggling were considered not meaningful.

##### Step 2: code each unit for novelty

Next, all turns were coded on one of the four levels of novelty. In Table 4 below, the first part of the coded transcript is presented, along with explanations for each given code.

**Table 4:** First 15 turns of transcripts and codes for the problem-solving task.

**Turn**	**Transcript**	**Level novelty**	**Motivation**
1	*T: So, you connected the two syringes. Can you explain how we can make this red mark inside go to here?*		
2	S: You do like this… [pulls]	3	Student refers to pulling, which is new.
3	*T: Yes, what will happen?*		
4	S: When you push down, the wind will go to there, and then to there. (points finger).	3	Student mentions pushing and wind moving through the tube, which are both new
5	*T: The wind will go to there to here?*		
6	S: And then it will blow up.	1	Small elaboration because blowing up is the target of the task, she mentions the consequence of the action described above.
*7*	*T: Blow up? To where?*		
8	S: To here. (points at red mark)	1	Small elaboration because she just adds exact location, which was the task assignment.
9.	*T: Now we will try it. One, two, three*.		
10.	S: (Pushes syringe to the red mark)	0	No new elements because pushing was mentioned.
11.	*T: Great job. Can you explain to me what happened?*		
12.	S: It went up.	0	No new elements.
13.	*T: Yes exactly, and what made that happen? What made it go to the red mark?*		
14.	S: Because the wind was… the wind was blowing that up.	0	No new elements, “wind” and “blow up” where already mentioned before.
15.	*T: Okay, because the wind blew this up. Great*.		
16.	S: I like this game.	3	Affective aspect of task was not mentioned before and is therefore new, but is slightly less appropriate in this task context.
17.	S: (pushes the syringe)	0	Repetition of action

*S, Student; T, Teacher. Teacher turns are in italics. (…) marks non-verbal turns*.

##### Step 3: code each unit for appropriateness

In the case of Sarah, all units were highly appropriate. The child was clearly immersed with the task and all actions and verbalizations in the 4-min fragment were related to task elements. There were several slight deviations, in which Sarah expressed that she liked the task. As these utterances did not contain a description, prediction, or explanation, and did not refer to specific task elements either, we decided to score them at level 2 on the appropriateness scale.

## Possibilities for data analysis

### Time series

**Figure 2:**
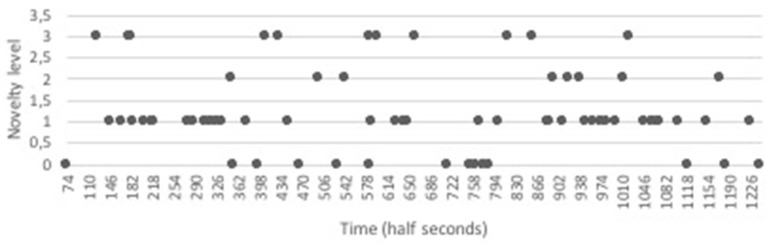
Time series of Novelty levels for John.

**Figure 3:**
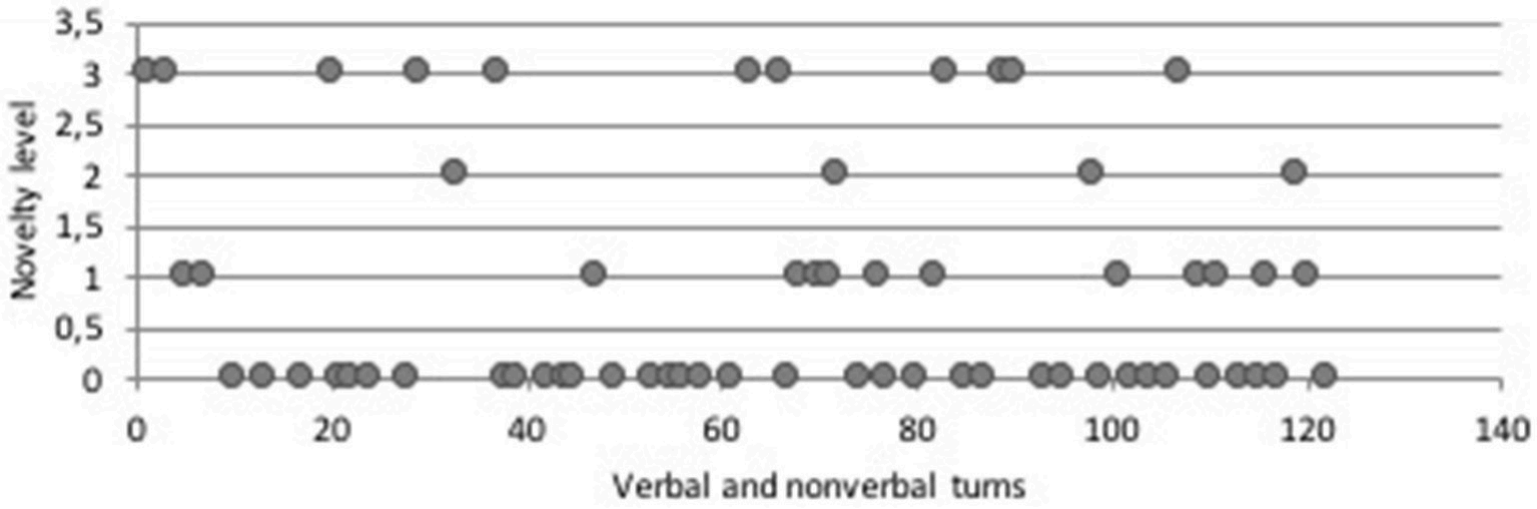
Time series of Novelty levels for Sarah.

A first inspection of the data can be obtained by plotting the levels of novelty and appropriateness over time. In Figure 2, the levels of novelty in the case of John were plotted over time. Looking at this graph, we can see that John frequently switches between different novelty levels. Both at the beginning of the assignment (between time point 108 and 330) and later on (time point 856 to 1077), we see an episode where a new idea (level 3) is followed by a dense series of small elaborations (level 1). This seems to be quite characteristic for this student working on this task. The time series of Sarah's novelty levels is shown in Figure 3. Here it is clearly visible that Sarah also frequently switches between different novelty levels. In Sarah's task behavior, we also observe that high levels of novelty occur across the session, and often alternate with less novel ideas or actions. Between turn 40 and turn 60 there seems to be a temporal “dip” in her creative behavior, with many repetitions of the same idea. After time point 60, actions and ideas with a relatively high level of novelty re-emerge (and when observing the video, it becomes clear that point 60 is exactly when a new task element is introduced, in the form of the Y-shaped tube).

### State space grids of novelty and appropriateness

After having coded the two dimensions of creativity, we can now combine them. One particularly useful technique when analyzing the interactions between both dimensions is the *State Space Grid (SSG) method* (Hollenstein, 2013). This technique is based on the idea that combinations of behaviors can be described in terms of their movements across the range of all behavioral possibilities for a given system. The data are described according to two ordinal variables that define the behavior of interest—in this case the novelty and the appropriateness dimension. The child's actions are seen as a *state* of the system, and they are represented by dots. Consequently, all *movements* between states are presented by lines. The advantage of using SSGs is not only that it offers a powerful visual analysis of the behavior in qualitative terms, but also that the software computes a set of *measures* that express the global flexibility or stability of the child's repertoire as shown in a specific task setting (for an example of this, see “Further analyses”).

**Figure 4:**
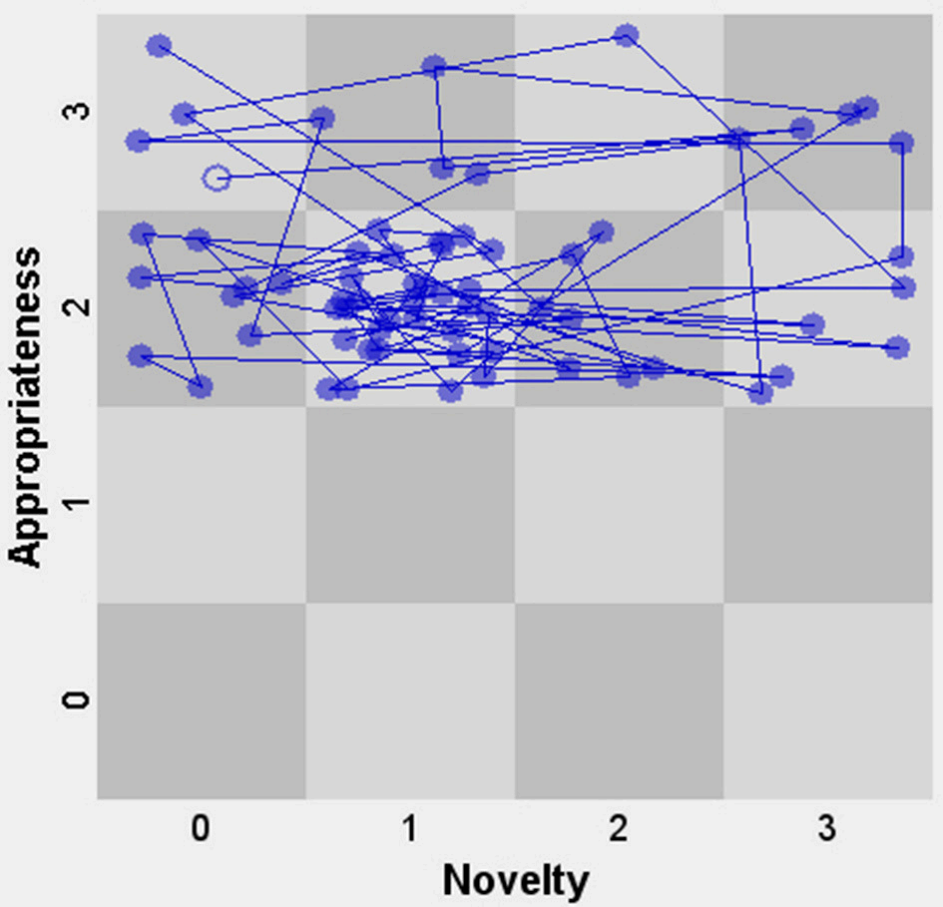
State space grid of Novelty and Appropriateness for John.

Figure 4 displays the SSG of novelty and appropriateness for John, with appropriateness on the vertical axis and novelty on the horizontal axis. Each node represents one unit or event. Since we coded units as discrete events (due to our choice to code utterances, turns and short-lived actions), the duration is not taken into account and all nodes are therefore the same size. For instance, a node in the bottom left corner represents an event that was both low in novelty and low in appropriateness (for the sake of visibility, the exact locations of nodes within a cell are random). The open node represents the first event. Overall, we see in Figure 4 that there is a cluster of ideas with a lower level of novelty (small elaborations) and a high level (2) of appropriateness. This corresponds with the observation that John is frequently engaged in a series of small elaborations on the same overarching idea, while staying focused on the task (for instance, the novel idea that a guitar should be added to the piece is followed by John trying out many different guitar loops before selecting one).

**Figure 5:**
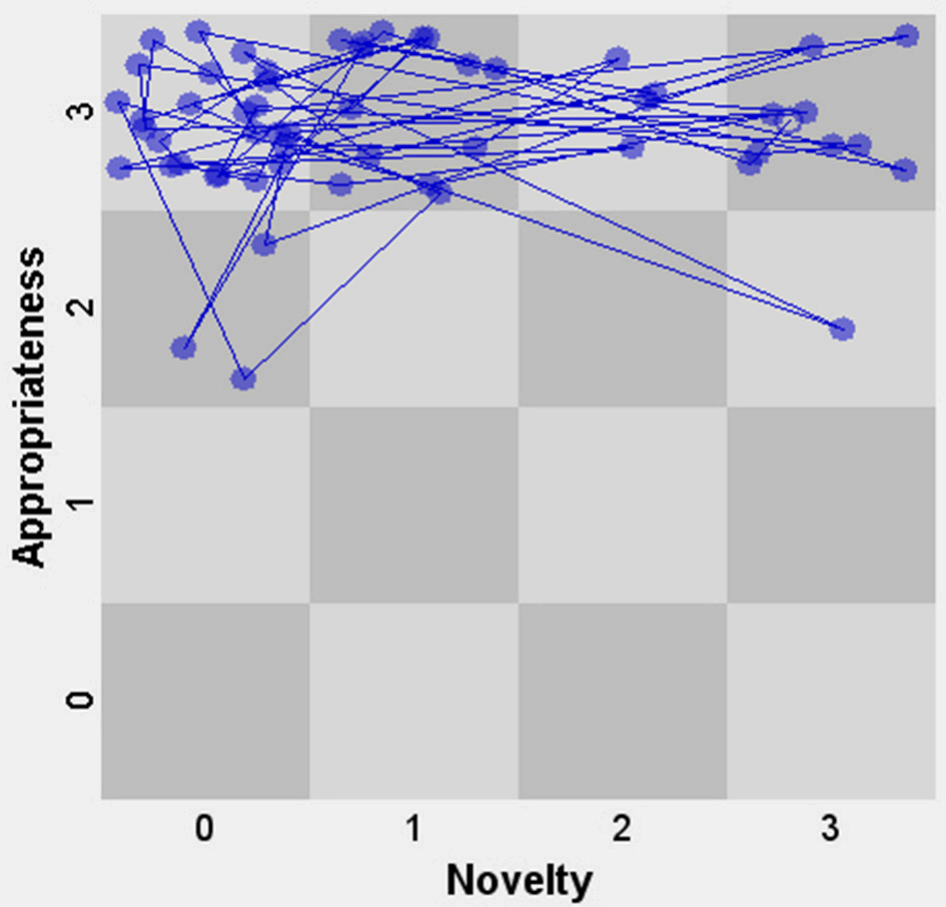
State space grid of Novelty and Appropriateness for Sarah.

It can be observed in Figure 5 (the SSG for Sarah) that there are many changes in the novelty dimension of the scale, with actions and ideas constantly moving from left to right and back. Appropriateness is much less variable in that regard. However, three out of four instances that show that the child drops slightly in appropriateness occur when novelty is also low.

So far, we have seen in both illustrations that the assignment of the codes could be done in a relatively straightforward way. The resulting analyses showed that in each case the actions and verbalizations were quite variable regarding the novelty dimension. We also observed in both cases that novel elements were often introduced by the child after repeated turns with no or low levels of novelty. A clearly observable difference between the tasks was that, in comparison with the syringes task, the music task elicited more behaviors that were variations on previous actions than the syringes task. An open-ended task like a music composition might involve more new “big ideas” that are then further developed through elaborations on those ideas. Although in both cases the task led to many highly appropriate actions and verbalizations, the music task elicited more level 2 actions and fewer level 3 actions than the syringes task. The reason for this is that the music assignment is more demanding, as it involves relating music to a storyline—compared to simply connecting task elements, such as in the syringes task.

### State space grids of novelty and teacher behavior

**Figure 6:**
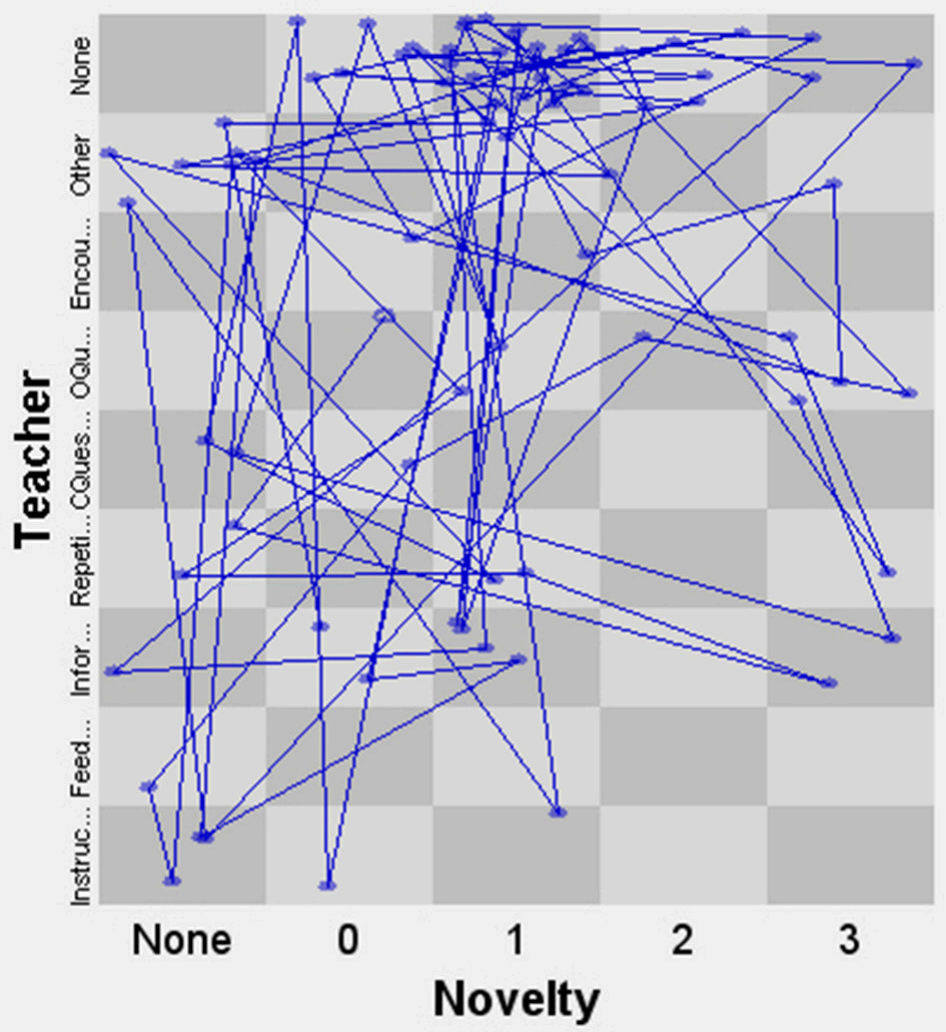
State space grid of Novelty and Teacher behavior for John.

**Figure 7:**
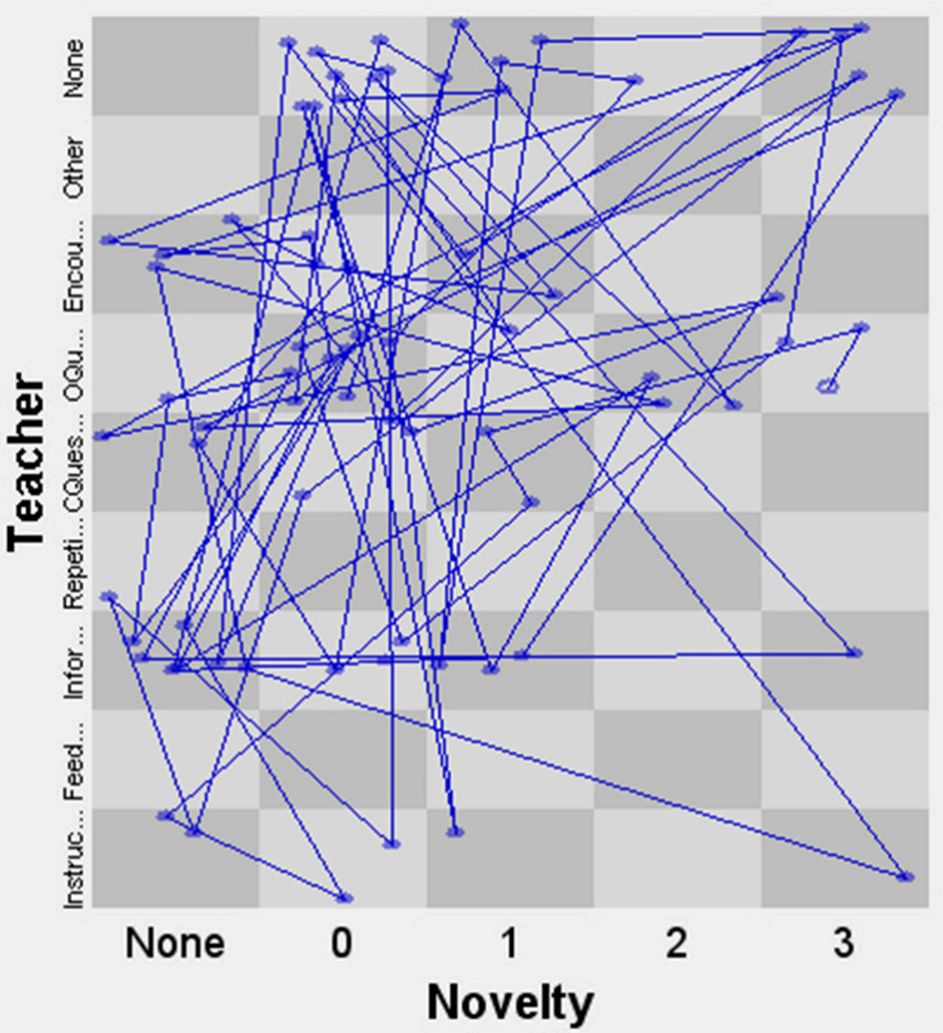
State space grid of Novelty and Teacher behavior for Sarah.

In order to further demonstrate the potential of the micro-developmental measure of creativity, we will show how it can be combined with another real-time variable. In the context of this study, we could for instance relate it to the utterances of the teacher—which is what we will do in the next example. In this case, we chose to code each teacher utterance into one of the following categories: Instruction, Feedback, Information, Repetition, Closed Question, Open Question, Encouragement, None, or Other. The categories were ordered according to the underlying dimension of how much room the teacher leaves for student initiative in each case (based on previously developed and validated scales of “openness” Meindertsma, 2014 and “autonomy support” Kupers, 2014). For instance, giving directive instructions leaves less room for student initiative than asking an open-ended question or providing encouragement. The advantage of linking these two variables is that it is possible to use the SSG-technique to plot the interactions on both dimensions. We show the application of this in Figures 6, 7 (these state space grids represent, respectively, the interactions of John and Sarah during the music and linked syringes tasks described above). Each blue dot in the graph represents a teacher utterance followed by a student action. For instance, a dot in the bottom left corner means that the teacher gave an instruction that was followed by a student turn with the lowest level of novelty (0). The last category on both axes, None, indicates a student turn that was not preceded by a teacher utterance (in other words, a student self-iteration) or vice versa (a teacher self-iteration). In this way, we can analyze which teacher utterances are followed by higher or lower levels of student novelty. Furthermore, we can also inspect whether the dyadic interaction is characterized by strong attractor states or high variability over time and across states. These applications are similar to what is used in Menninga et al. (2017) and van Vondel et al. (2016), but in this case they feature a measure of the student's creativity instead of the student's level of cognitive performance.

In Figure 6, we see that although the data of John are scattered broadly across the grid, most data points are in the top half of the grid. The amount of student self-iterations shows that quite often there are sequences where the student proposes ideas without a specific prompt from the teacher, indicating that the creative process is—at times, at least—student-led. We also see teacher utterances with a high level of openness, which lead to student turns with varying degrees of novelty. As for the interaction between Sarah and her teacher in Figure 7, we observe a lot of variability in both teacher behavior and the novelty of student responses. Though most interactions lead to relatively low-novelty responses, the responses that are high in novelty seem either to be preceded by an open question or to be a self-iteration.

In both cases, there are no clear attractor states in the interaction dynamics between the children and the teachers. The quantitative measurements show a high level of variability over time, especially for Sarah. Dispersion, which can vary between 0 (all events in one state) and 1 (all events spread out evenly over the grid), was 0.91 for John and 0.96 for Sarah—suggesting that Sarah's interactions may be slightly more variable than John's.

This illustration shows that combining the micro-developmental coding of creativity with a second variable (such as teachers' verbalizations) offers many possibilities for analyzing their dynamic interactions on a more advanced level. These interactions can be visually inspected and quantified by means of the measures offered by the technique. These measures can be used to make, for instance, a comparison between different teacher-student dyads working on the same task, or on different (versions of) tasks, etc. Another option is to analyze the interactions between peers as they work together on a task, investigating how creative behaviors emerge from their collaboration.

## General discussion

Creativity research has flourished in the last decades. When it comes to empirical research, creativity is mostly measured either at the level of the person (by means of divergent or creative thinking tests) or at the level of the product (by means of consensual assessment) (Kupers et al., submitted). Studies on creative processes are usually purely qualitative. These qualitative studies provide thorough descriptions of creative processes in a certain domain (such as dance, music, or scientific research), but their domain specificity makes it hard to generalize their findings to other contexts. For this reason, our aim was to develop a quantitative measure of creativity that on the one hand is focused on measuring creativity in the here-and-now of the creative process, and on the other hand is systematic and generic in the sense that it can be applied to many different contexts. We have illustrated the potential of this micro-developmental measure by applying it to an open-ended musical composition task as well as a closed-ended scientific reasoning task.

The framework we propose is rooted in the sociocultural tradition of studying creativity most prominently represented by Csikszentmihalyi and his Systems model of Creativity (Csikszentmihalyi, 1988), and since then developed by Sawyer (1999, 2007) and Glǎveanu (2010a,b, 2014), amongst others. The micro-developmental nature of the framework allows us to zoom in on the *interaction* between the person being studied, other persons, and the creative product or task (Glǎveanu, 2013b). This is in line with a recent movement within psychology—originating from cultural, ecological perspectives and Complex Dynamical Systems approaches—toward reinterpreting psychological constructs as dynamic, embodied, embedded and enacted (Granic, 2005; Lichtwarck-Aschoff et al., 2008; Rowlands, 2010; Borsboom and Cramer, 2013; de Ruiter et al., 2017). These new theoretical developments ask for new approaches to measuring creativity as well, and our instrument forms an important step in further developing these ideas. Central to a process approach to creativity is the idea that creativity emerges from moment to moment in interaction between a person, the immediate social environment (teachers, peers etc.), and the task (Glǎveanu, 2013b; Kupers et al., submitted). However, one domain that remains relatively unattended in creativity research is that of the task. From a dynamic, enactment perspective, any task has certain affordances. Affordances are characteristics of the task that provide opportunities in the interaction with that task (Gibson, 1977; Withagen et al., 2012, 2017). For instance, a task that requires children to copy a drawing by their teacher provides very little opportunity for students to come up with their own ideas, while the assignment to design and draw your own dream house gives students much more opportunities to come up with new ideas. With the framework we present in this article, it is possible to look in detail at creative affordances of different kinds of tasks in many different settings.

Our coding framework is based on the two core components of creativity: novelty and appropriateness. Although the importance of both elements is underlined theoretically (e.g., Cropley, 2006), psychological tests of creativity usually only assess “divergent thinking,” which is basically the ability of a person to come up with many (fluency) ideas that are original (novelty) and unrelated to each other (flexibility). The more novel, unrelated and appropriate an idea is, the greater is its underlying trait of creative thinking—that is the assumption of these tests. The question is whether “more is better” also applies to measuring the creative process, which our coding frame aims to address. Is a creative process more “successful” if it features more ideas with the highest level of novelty, given a high level of appropriateness? More research is necessary—especially on the micro level—to unravel the ways in which appropriateness and novelty interact from moment to moment.

## Limitations and recommendations for future research

This paper presents a general framework for the coding of creativity on the level of micro-development, in the interaction between a person, task, and the direct social environment. While an advantage of the proposed method (and an aim of the authors) was that the instrument is applicable to many different contexts, this also poses limitations. We have stressed throughout this paper that, for each dataset, the general coding framework presented here needs to be adjusted in order to form an actual coding *scheme*—which involves specific decision rules regarding the segmentation of the data in units of analysis and the coding of those units. Though we have provided an illustration of how to do this in the case of two different creative tasks, this should not be seen as an attempt to validate the method but rather as a demonstration of applying the coding frame to specific data—which is an important first step. Any coding schemes that future researchers construct on the basis of our general coding framework need to be validated on larger datasets, as is generally the case with observational coding schemes.

Important theoretical foundations of this coding framework have been social-constructivist approaches to creativity (Csikszentmihalyi, 1988; Amabile, 1996; Sawyer, 2007; Glǎveanu, 2010a). This automatically raises two questions: a. Is the coding scheme only applicable to creativity in social interactions, as demonstrated here? b. When the coding scheme is indeed applied to social interactions, how should the actions of the “other” (in this case, the teacher) be coded? With regard to the first question: the coding scheme can also be applied to individual creativity (of children as well as adults), as long as all the steps in the creative behavior are observable. In order to get a better understanding of individual creativity as an emergent property from real-time interactions, it could be interesting to follow individuals over longer periods of time as they engage in different creative tasks. With regard to the second question: we have provided an example of how teacher behaviors can be coded, but many different options are possible. In peer interactions, it is possible to code novelty and appropriateness of both interaction partners. In teacher-student interactions, one promising construct is “teaching for creativity.” By translating this construct into observable behavior, we can get a direct analysis of which aspects of the theoretical construct actually lead to student creativity.

## Ethics statement

This study was carried out in accordance with the recommendations of the ethical guidelines of the University of Groningen; respectively, the Ethical Committee of Pedagogical and Educational Sciences and the Ethical Committee of Psychology. The protocols were approved by the Ethical Committee of Pedagogical and Educational Sciences and the Ethical Committee of Psychology (University of Groningen). All (parents of) subjects gave written informed consent in accordance with the Declaration of Helsinki.

## Author contributions

EK designed method, discussed application of method with MVD and AL-W, wrote first draft of article (and took the lead in further drafts), collected and analyzed data for the first case study. MVD co-designed method, wrote parts of the first and second draft, gave comments on several drafts, analyzed data for the second case study. AL-W co-designed method, gave comments on analyses data and several drafts.

### Conflict of interest statement

The authors declare that the research was conducted in the absence of any commercial or financial relationships that could be construed as a potential conflict of interest.
